# Overcoming Perfectionism: Protocol of a Randomized Controlled Trial of an Internet-Based Guided Self-Help Cognitive Behavioral Therapy Intervention

**DOI:** 10.2196/resprot.6378

**Published:** 2016-11-11

**Authors:** Radha Kothari, Sarah Egan, Tracey Wade, Gerhard Andersson, Roz Shafran

**Affiliations:** ^1^ Division of Psychology and Language Sciences University College London London United Kingdom; ^2^ School of Psychology and Speech Pathology Faculty of Health Sciences Curtin University Perth Australia; ^3^ School of Psychology Flinders University Adelaide Australia; ^4^ Department of Behavioural Sciences and Learning Linkoping University Linköping Sweden; ^5^ Department of Clinical Neuroscience Karolinska Institute Stockholm Sweden; ^6^ Institute of Child Health Population, Policy and Practice Programme University College London London United Kingdom

**Keywords:** perfectionism, cognitive behavioral therapy, randomized controlled trial, Internet-based intervention, anxiety, depression

## Abstract

**Background:**

Perfectionism is elevated across, and increases risk for, a range of psychological disorders as well as having a direct negative effect on day-to-day function. A growing body of evidence shows that cognitive behavioral therapy (CBT) reduces perfectionism and psychological disorders, with medium to large effect sizes. Given the increased desire for Web-based interventions to facilitate access to evidence-based therapy, Internet-based CBT self-help interventions for perfectionism have been designed. Existing Web-based interventions have not included personalized guidance which has been shown to improve outcome rates.

**Objective:**

To assess the efficacy of an Internet-based guided self-help CBT intervention for perfectionism at reducing symptoms of perfectionism and psychological disorders posttreatment and at 6-month follow-up.

**Methods:**

A randomized controlled trial method is employed, comparing the treatment arm (Internet-based guided self-help CBT) with a waiting list control group. Outcomes are examined at 3 time points, T1 (baseline), T2 (postintervention at 12 weeks), T3 (follow-up at 24 weeks). Participants will be recruited through universities, online platforms, and social media and if eligible will be randomized using an automatic randomizer.

**Results:**

Data will be analyzed to estimate the between group (intervention, control) effect on perfectionism, depression, and anxiety. Completer and intent-to-treat analyses will be conducted. Additional analysis will be conducted to investigate whether the number of modules completed is associated with change. Data collection should be finalized by December 2016, with submission of results for publication expected in mid-year 2017. Results will be reported in line with recommendations in the Consolidated Standards of Reporting Trials Statement for Randomized Controlled Trials of Electronic and Mobile Health Applications and Online TeleHealth (CONSORT-EHEALTH).

**Conclusions:**

Findings will contribute to the literature on treatment of perfectionism, the effect of treating perfectionism on depression and anxiety, and the efficacy of Internet-based guided self-help interventions.

**ClinicalTrial:**

ClinicalTrials.gov NCT02756871; https://clinicaltrials.gov/ct2/show/NCT02756871 (Archived by WebCite at http://www.webcitation.org/6lmIlSRAa)

## Introduction

### Overview

Being a perfectionist has both positive and negative connotations. In day-to-day life, a moderate level of perfectionism is commonly associated with success and achievement, but perfectionism has been found to be elevated across, and associated with, a range of psychiatric disorders, including major depression, generalized anxiety disorder, obsessive compulsive disorder, bipolar disorder, posttraumatic stress disorder, panic disorder, eating disorders, body dysmorphic disorder, social anxiety disorder, chronic fatigue syndrome, and suicidal ideation and behavior [1,2]. Perfectionism has been found to increase risk for, and contribute to, the maintenance of eating disorders, anxiety disorders, and depression [3] and has been identified as a key maintenance factor in the transdiagnostic model and cognitive behavioral treatment of eating disorders [4]. Perfectionism can impede treatment progress and lead to poorer outcomes in treatment of patients with chronic pain [5], depression [6], and anorexia nervosa [7,8]. Perfectionism has also been associated with poor physical health and premature death [9,10].

**Figure 1:**
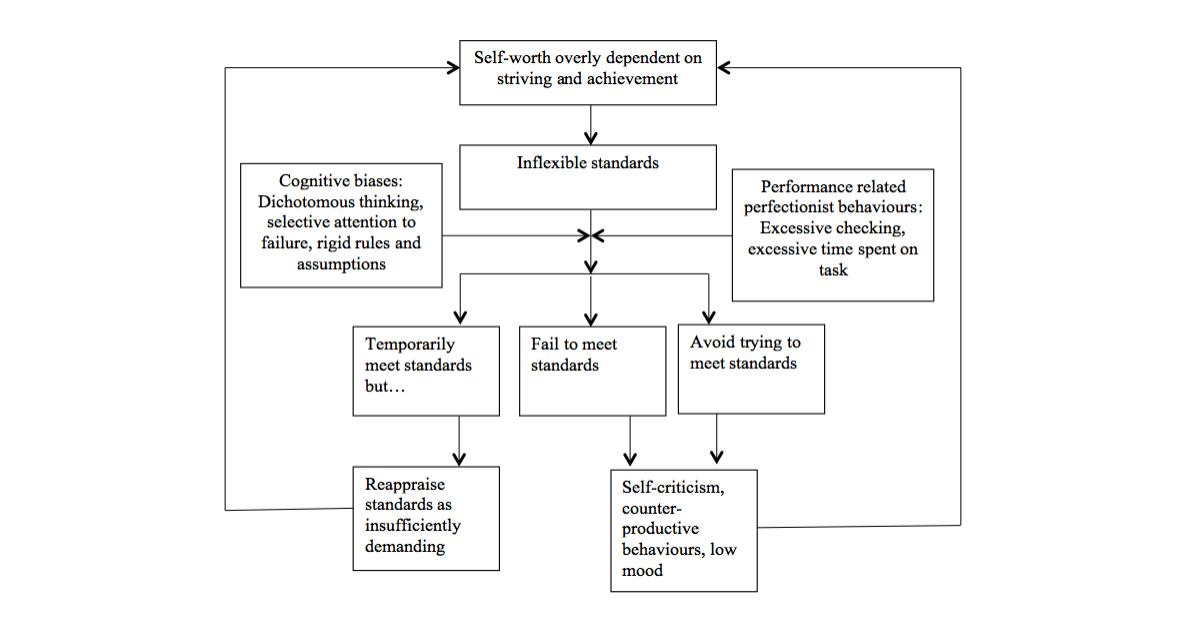
The cognitive behavioral model of clinical perfectionism, reproduced from Cognitive Behavioural Treatment of Perfectionism [2].

The problems associated with perfectionism highlight the need for intervention, and with this in mind a cognitive behavioral maintenance model of perfectionism was developed by the Oxford Centre for Research on Eating Disorders, led by Christopher Fairburn. This cognitive behavioral analysis, particular to a specific form of perfectionism typically seen in clinical settings, characterizes *clinical perfectionism.* According to the cognitive behavioral account, people with clinical perfectionism are determined in the pursuit of their own personally demanding, rigid standards in at least one salient domain. These standards are pursued regardless of adverse consequences due to the individual’s self-evaluation being almost entirely reliant on achievement and striving; individuals develop rigid standards and rules which come to dominate their lives, often expressed as “musts” and “shoulds,” and continually strive for achievement and success to avoid the occurrence of their feared outcome, perceived failure. This in turn leads to behaviors that maintain clinical perfectionism such as avoidance, procrastination, repeated checking, and excessive thoroughness. The cognitive behavioral account goes on to suggest that an individual’s assessment of whether they have met their standards is subject to well-established cognitive biases including dichotomous thinking, attention to negative rather than positive feedback, and discounting of success. This means that people with clinical perfectionism are likely to perceive themselves as having failed to meet their standards, leading them to be self-critical; experience emotional arousal at the thought of failure, anxiety, and low mood; and engage in further counterproductive behavior such as increased checking and thoroughness [11,12] (see Figure 1). For a full explanation and evaluation of the cognitive behavioral maintenance model of clinical perfectionism see Shafran et al [13].

### Cognitive Behavior Therapy for Clinical Perfectionism

Based on the cognitive behavioral maintenance model of clinical perfectionism, a cognitive behavior therapy (CBT) protocol has been developed consisting of 10 modules typically delivered over 8 weeks. In line with the principles of CBT, emphasis is on maintenance over etiology. Cognitive behavioral techniques are used to challenge the cognitive biases, personal standards, and self-criticism that maintain clinical perfectionism and to broaden the client’s attention and scheme for self-evaluation. Full details of CBT for clinical perfectionism can be found in the published manual [2].

There is a growing body of evidence to support the efficacy of using CBT to target perfectionism. A recent meta-analysis of 8 studies showed that CBT was effective at reducing symptoms of perfectionism, anxiety, and depression, with medium-to-large pre-post intervention effect sizes according to Cohen’s [14] criteria for change [15]. Since publication, a further 4 studies have been added to the meta-analysis, showing large effect sizes for the reduction of symptoms of perfectionism and large effect sizes for the reduction of psychological disorders (Lloyd et al, unpublished thesis).

### Internet-Based Self-Help Cognitive Behavior Therapy for Perfectionism

Given the increased need and desire for Internet-based interventions, an Internet-based version of CBT for perfectionism (where participants work independently without guidance from a therapist) has been developed and tested. An initial study found that Internet-based CBT for perfectionism led to significant decreases in perfectionism, anxiety, and depression [16]; however, a more recent study has found that although this Internet-based intervention led to a reduction in symptoms of perfectionism, it was less effective than the face-to-face version of the treatment in maintaining this change at a 6-month follow-up [17]. In addition, the Internet-based intervention did not affect levels of depression and anxiety.

Computer and Internet-based interventions are commonly associated with a number of advantages over face-to-face treatment, such as reduced cost to health care providers, increased patient anonymity, and increased convenience for the patient with regard to time and location of treatment [18-22]. Furthermore, the demand for psychological therapies outstrips availability, and Internet-based intervention can help fill this gap. However, Musiat and colleagues [21] found that individuals are more likely to use face-to-face therapy than Internet-based interventions. The authors of this study suggest that this might be due to the personal support that participants associate with face-to-face therapy, noting that personal support was rated the second most important factor when considering seeking help for mental health disorders, after the helpfulness of an intervention [21].

Being able to process thoughts and feelings in written form and developing a virtual relationship with a therapist were shown to be important to mental health users when receiving CBT delivered online by a psychologist [23]. A growing body of evidence suggests that Internet-based self-help interventions would benefit from a personal component of this nature, and Internet-based treatment of depression and anxiety has in fact been found to benefit from therapist guidance, leading to improved recovery rates and lower dropout rates [18,24]. A meta-analysis of randomized controlled trials (RCTs) of computer-based interventions for depression found that studies which included therapist support, or support from nonclinically trained guides, had medium effect sizes (*d*=0.78 and *d*=0.58, respectively), while the effect size for interventions without support was small (*d*=0.36) [25]. Given that a goal of Internet-based interventions is to increase availability, the effect size observed for nonclinically trained guides is encouraging, suggesting that guidance could be provided by individuals with minimal training. A more recent meta-analysis found a correlation between level of support and effect size of treatment, comparing interventions with no human contact; contact before the intervention only; contact mainly during the intervention; and contact before, during, and after the intervention. Average effect sizes were found to be 0.21, 0.44, 0.58, and 0.76, respectively [26]. This study aims to investigate whether additional guidance and feedback in the form of email (guided self-help) might also improve the effectiveness of an Internet-based version of CBT for perfectionism.

## Methods

### Setting and Intervention

This study is an RCT of an Internet-based guided self-help intervention for clinical perfectionism called *Overcoming Perfectionism* [27]. Recruitment, interventions, and measurement will take place online and through the website. This Internet-based version of the treatment has been adapted from the manual on perfectionism-specific CBT, *Cognitive Behavioural Treatment of Perfectionism* [2]. Content was made briefer and more focussed for the Internet-based intervention, and video was used. The intervention consists of 8 modules based on CBT techniques (see Table 1), and participants will be allowed 12 weeks to complete it and be provided with guidance and support.

Participants will still be given access to the intervention after this 12 weeks, but guidance will not be provided. Each module begins by providing psychoeducation and examples and then allows the participant to answer questions and complete relevant worksheets to create an idiosyncratic model of their own unhelpful perfectionism. In line with the principles of CBT, participants are encouraged to integrate their learning into their day-to-day lives by completing thought records, challenging cognitions, conducting surveys, and performing experiments in the form of between-session work.

Completed worksheets can be viewed by a guide who has been allocated to the participant and who is able to provide feedback and suggestions to the participant in the form of Internet-based written communication. Participants are also able to communicate directly with their allocated guide through the website, allowing them to ask questions or respond to the feedback that they have received.

### Guidance and Feedback

Guidance and feedback will be provided by psychology undergraduates, graduates, master’s students, PhDs, and trainee clinical psychologists. Guidance will be based upon the manual *Cognitive Behavioural Therapy of Perfectionism* [2], which guides will read as part of their training and refer back to when giving guidance. All guides will be provided with a range of sample responses for each module which they will study as part of their training and refer back to when providing feedback.

Guides will receive supervision throughout in 2 forms. First, all guidance and feedback written by guides will be checked by a qualified clinical (RS) or research psychologist (RK), who will help the guides develop their responses and consider different ways to support and encourage participants. In this way all participants will receive feedback that has been contributed to by at least 2 guides. This process will serve to provide guides with continuous training and supervision throughout and also to keep responses to participants consistent. The second aspect of supervision will be in the form of monthly supervision meetings during which guides will be supported with their case management and be able to discuss complex cases and challenges they may be experiencing as guides. The content of the feedback will be as closely aligned to the content of the therapy manual as possible [2].

Recommendations in the literature highlight a number of mechanisms of action through which feedback is effective, and these will be adopted when drafting feedback for participants [28]:

1. Guides will summarize and reflect information, thoughts, and experiences provided by participants, enabling participants to process their thoughts and feelings and reflect upon their experiences [29].

2. Feedback that is personally relevant is more likely to lead to deeper processing and is therefore more likely to be examined for its content [30]. Addressing recipients by their name is thought to sufficiently personalize feedback, but in addition to this guides will refer to specific experiences and examples that have been provided by participants when responding [31].

3. Cognitive theories highlight the importance of providing information that will support participants in changing their knowledge, thinking, and behavior, particularly if participants have misunderstood or mistaken elements of the intervention [32]. Guides will directly address the thought challenging, behavioral experiments, and other cognitive behavioral tasks that participants engage with to support them in thinking about the impact of the changes made and the potential for transferring their new skills to other situations. Guides will also support participants in the design of behavioral experiments so that participants gain the maximum benefit from challenging their behavior.

4. Adopting the principles of motivational interviewing can make personalized feedback effective in strengthening motivation for change [33]. Guides will remind participants of their goals and personal motivations for change, emphasizing the discrepancy between where they are and where they would like to be with regard to problematic behaviors and how much progress they have made since the start of the intervention, so as to support participants and strengthen continued engagement.

Participants will receive feedback and guidance as they complete each module and submit the relevant worksheets, with the average length of feedback for each worksheet being 1 to 2 paragraphs. Participants will also receive guidance if they specifically request help in understanding or completing modules and between session work.

**Table 1:** Modules and components of Overcoming Perfectionism, an Internet-based guided self-help intervention for perfectionism.

Module	Module Components
1. Understanding Perfectionism	1.1. What is unhelpful perfectionism? 1.2. Why perfectionism continues 1.3. Fact or fiction? 1.4. “The harder you work, the better you'll do” Fact or fiction? 1.5. Facts about perfectionism and performance 1.6. Preparing for change 1.7. Key take away 1.8. Between-module work
2. Your Perfectionism Cycle	2.1. Between-module work 2.2. A reminder 2.3. The first steps 2.4. Drawing your own diagram 2.5. Between-module work 2.6. Take-home message
3. Surveys and Experiments	3.1. Between-module work 3.2. Perfectionism behaviors 3.3. Surveys 3.4. Reflect on the responses 3.5. Behavioral experiments 3.6. Different forms of behavioral experiments 3.7. An added benefit 3.8. Between-module work 3.9. Take home message
4. New Ways of Thinking	4.1. Between-module work 4.2. Changing thinking 4.3. Imagining vivid future positive outcomes 4.4. From all or nothing thinking to flexibility and freedom 4.5. “Rules break, guidelines bend:” Turning rigid rules into guidelines 4.6. Changing thinking styles 4.7. Between-module work 4.8. Key take away
5. Useful Skills for Managing Unhelpful Perfectionism	5.1. Procrastination 5.2. Problem-solving 5.3. Pleasant events 5.4. Take home message 5.5. Before the next module
6. Self-Criticism or Self-Compassion	6.1. How to respond 6.2. Take home message 6.3. Before the next module
7. Reexamining the Way We Examine our Self-Worth	7.1. Your self-worth 7.2. Step 1. Recognizing that your self-worth can be independent of your achievements 7.3. Step 2. Encouraging flexible and realistic goals 7.4. Step 3. Spreading your self-worth across as many areas of your life as possible 7.5. Step 4. Develop more balance in what you pay attention to daily 7.6. Take home message 7.7. Before the next module
8. Staying Well—Managing Unhelpful Perfectionism in the Long-Term	8.1. Improve your sense of self-worth 8.2. Questions 8.3. Thank you!

### Participants

Participants will be recruited through university notice boards and online platforms, recruitment websites such as www.callforparticipants.com, and social media platforms such as Facebook and Twitter. Researchers will not approach potential participants directly, but rather interested individuals will be directed to the study website where they will be able to find out more about the study, read the information sheet, and give consent for participation. After registering online participants will complete screening measures, responses to which will determine their eligibility for inclusion. To be eligible for inclusion in this study, participants must be 18 years or older, fluent in English, and score 1 standard deviation above published norms on the “Concern over mistakes” subscale of the Frost Multidimensional Perfectionism Scale [34] (ie, a score of ≥29 [35]). Participants will be excluded if they report suicidal thoughts or intent. In this case participants will be telephoned by a clinical psychologist to be assessed for risk and signposted to the relevant services.

Participants with elevated levels of psychopathology will not be excluded from the study due to the established comorbidity between psychopathology and clinical perfectionism. Upon registering and providing informed consent, participants will be asked to complete a collection of screening measures to determine their eligibility for the study. If eligible, participants will be randomly allocated to the experimental group to complete the intervention or the control group (no intervention). Randomization of participants will be performed by a third party, unconnected to the study, who will create a randomization schedule using a Web-based randomizer [36]. Guides will be paired with participants who have been allocated to the treatment group after randomization. Participants who do not meet criteria for inclusion in the study will be sent a copy of *Overcoming Perfectionism: A Self-Help Guide Using Cognitive Behavioural Techniques* [11] and will be signposted to other services.

### Sample Size

An a priori power calculation was conducted using a tool designed by Hedeker and colleagues which is appropriate for determining power for longitudinal designs [37], with a 2-tailed alpha of .05, 3 assessment points, a pre-post correlation for the primary outcome measure (“Concern over mistakes” subscale) of 0.61, and attrition rates of 50%. Both the pre-post correlation and expected attrition rate were based upon a similar RCT of a Web-based intervention for perfectionism [17]. A sample size of 40 enrolled participants per group, with 20 participants completing per group, would provide 80% power at 2-sided *P*<.05 to detect large effect size (0.80) difference between the control and intervention groups. This use of a large effect size was also based upon the previous RCT conducted by Egan and colleagues [17].

### Measures

#### Overview

Self-report questionnaire measures of perfectionism, anxiety, and depression will be collected from all participants at 3 time points: (1) prior to any intervention as a baseline, (2) 12 weeks after the participant has been randomly assigned to the treatment or control group (to assess change), and (3) 24 weeks after being randomly assigned to the treatment or control group (to assess whether change is maintained at follow-up).

Participants in the experimental group will also complete a measure of clinical perfectionism weekly to monitor progress. Engagement with the website will be monitored through questions asking participants how much time they spent completing each module and the between session tasks.

#### Measures of Perfectionism

The Frost Multidimensional Perfectionism Scale (FMPS) self-report measure consists of 35 items grouped into 6 subscales: Concern over mistakes (eg, “I should be upset if I make a mistake”), Doubts about actions (eg, “I usually have doubts about the simple everyday things I do”), Personal standards (“I set higher goals than most people”), Parental expectations (“My parents set very high standards for me”), Parental criticism (“My parents never tried to understand my mistakes”), and Organization (“I try to be an organized person”). Participants respond on a 5-point scale ranging from 1 = “strongly disagree” to 5 = “strongly agree.” The measure has been found to be both reliable and valid for use with nonclinical and clinical populations [34,38,39]. Participants will be considered eligible for inclusion in the study if they score 1 standard deviation above published means on the “Concern over mistakes” subscale (ie, a score of ≥29) [35]. This subscale is being used as the baseline and main outcome measure in line with previous RCTs investigating treatment of perfectionism [15,17]. The FMPS has been amended to reflect participant experience over the past month allowing us to measure change.

The Clinical Perfectionism Questionnaire self-report measure consists of 12 items reflecting participant experience over the past month (eg, “Have you pushed yourself really hard to meet your goals?” and “Have you raised your standards because you thought they were too easy?”) [40]. Participants respond on a 4-point scale ranging from 1 = “not at all” to 4 = “all the time.” This measure of clinical perfectionism was created by Fairburn, Cooper, and Shafran at the University of Oxford and has been found to have good reliability and validity in 2 community samples and an eating disordered sample [41]. The original version of this measure excluded perfectionism in the domain of eating, shape, and weight due to the design of the study in which it was developed, but for this study it has been amended to allow for perfectionism in this domain. As well as being administered at baseline, posttreatment, and at follow-up, a version of the measure amended to reflect participant experience over the past week will be administered to the treatment group weekly allowing us to monitor change.

#### Measures of Psychopathology

The Depression, Anxiety, and Stress Scales [42] is a 21-item self-report measure of depression, anxiety, and stress (eg, “I found myself getting upset by quite trivial things”) rated on a 4-point scale ranging from “Did not apply to me at all” to “Applied to me very much or most of the time.” It has been shown to be reliable and has been validated for use among clinical and community samples [42,43].

### Ethical Considerations

Ethical approval for this study has been granted by the University College London Research Ethics Committee (Project ID: 6222:001). Professor Roz Shafran is the primary investigator on the trial.

## Results

### Methodology

Data will be analyzed using T2 (postintervention and primary endpoint at 12 weeks) as the outcome variable adjusted for observations at T1 (baseline) in order to estimate the between-group (intervention, control) effect on perfectionism, depression, and anxiety. The follow-up effect of the intervention will be investigated in the same way with T3 (follow-up, 24 weeks) replacing T2 observations. Both completer and intent-to-treat (ITT) analyses will be conducted. ITT analyses will use multiple imputation to manage missing data. An analysis will be conducted to investigate whether the number of modules completed is associated with change. Results will be reported in line with recommendations in the Consolidated Standards of Reporting Trials Statement for Randomized Controlled Trials of Electronic and Mobile Health Applications and OnLine TeleHealth (CONSORT-EHEALTH) [44].

### Timeline

Ethical approval for this trial was granted in February 2015. Enrollment of participants began in July 2015. Data collection should be finalized by December 2016, with submission of results for publication expected in mid-year 2017.

## Discussion

### Overview

The aim of this study is to investigate whether CBT Internet-based guided self-help for perfectionism is effective at reducing perfectionism and symptoms of depression and anxiety. Perfectionism has been shown to be elevated across, and increase risk for, a range of psychological disorders, as well as having detrimental effects on day-to-day functioning directly. This is the first trial assessing the efficacy of an Internet-based self-help intervention for perfectionism that provides personalized feedback from a guide throughout. Outcomes will contribute to the literature on the treatment of perfectionism and providing guidance within Internet-based interventions.

### Limitations

Participants are only eligible for inclusion in the study if they score 1 standard deviation above the reported mean on the “Concern over mistakes” subscale, taken from a study investigating perfectionism within a student population [35]. A limitation of this study is that perfectionism may be relatively high among college students, making our cut-off for inclusion higher than necessary. Participants will also be excluded from the study if they report suicidal ideation or behavior; however, a limitation of the study is that due to ethical considerations we are not able to directly assess suicidal ideation or behavior and are reliant on participants volunteering this information to their guide. Participants reporting mental health difficulties other than perfectionism will not be excluded due to the established relationship between perfectionism and elevated levels of psychopathology. This may lead to an additional level of variability within the sample, and variation in psychopathology may impact treatment adherence and/or effectiveness. Randomization of participants will minimize these effects, however, and participants will also be asked to report any mental health diagnoses to investigate any potential differences between the treatment and control groups.

## Abbreviations

CBT: cognitive behavior therapy
FMPS: Frost Multidimensional Perfectionism Scale
ITT: intention-to-treat
RCT: randomized controlled trial
